# Exploring the Causal Relationship between Ibuprofen Use and Osteoarthritis Risk: A Mendelian Randomization Study

**DOI:** 10.3390/biology13090748

**Published:** 2024-09-23

**Authors:** Yongzhi Jian, Yanmin Lyu, Said Hashemolhosseini

**Affiliations:** 1 Institute of Biochemistry, Medical Faculty, Friedrich-Alexander-University of Erlangen-Nürnberg, 91054 Erlangen, Germany; 2 Division of Molecular and Experimental Surgery, Translational Research Center, Universitätsklinikum Erlangen, Friedrich-Alexander-University of Erlangen-Nürnberg, 91054 Erlangen, Germany; lyuyanmin@gmail.com; 3 Muscle Research Center, Friedrich-Alexander-University of Erlangen-Nürnberg, 91054 Erlangen, Germany

**Keywords:** ibuprofen, osteoarthritis, Mendelian randomization, GWAS, causal inference

## Abstract

**Simple Summary:**

This study investigated whether using the common painkiller ibuprofen increases the risk of developing osteoarthritis, a joint disease that causes pain and stiffness. We used two-sample Mendelian randomization, which helped us understand the relationship between drugs and disease by examining genetic data. We analyzed data from multiple studies to see if people with certain genetic markers linked to ibuprofen use were more likely to develop osteoarthritis. Our findings suggest that ibuprofen use may indeed increase the risk of developing osteoarthritis. This finding is important because ibuprofen, while effective in relieving pain, may have long-term effects on joint health. These insights may help doctors make better decisions about the use of ibuprofen in patients at risk of developing osteoarthritis.

**Abstract:**

This study explored the potential causal relationship between ibuprofen (IBU) use and the risk of developing osteoarthritis, a prevalent joint disorder characterized by pain and stiffness. We conducted a two-sample MR analysis using four distinct OA GWAS datasets as outcomes and single-nucleotide polymorphisms (SNPs) associated with IBU metabolism as exposures. The inverse variance weighted (IVW) and weighted median methods were utilized to assess the causal association by meta-analysis, while pleiotropy and heterogeneity were evaluated using MR–Egger regression and Cochran’s Q statistics. The MR analysis provided strong evidence for a causal association between IBU use and an increased risk of OA. A meta-analysis of the IVW and weighted median results across all datasets demonstrated an OR = 1.116 (95% CI = 1.063–1.170) and an OR = 1.110 (95% CI = 1.041–1.184). The consistency of the results obtained from different methods enhanced the reliability of the findings. Low pleiotropy and minimal heterogeneity were observed, further validating the results. The study supports a causal link between IBU use and an increased risk of OA, suggesting that IBU may accelerate the progression of OA while relieving symptoms. These findings highlight the importance of cautious use of IBU in clinical practice, especially considering its potential impact on long-term joint health.

## 1. Introduction

Osteoarthritis (OA) is a prevalent chronic joint disorder characterized by the degeneration of articular cartilage, leading to pain, stiffness, and functional impairment [1]. It has multiple risk factors, including genetic predispositions, obesity, joint injuries, and aging [2]. A study utilizing Mendelian randomization indicated that higher body mass index causally increases OA risk, emphasizing the role of obesity as a modifiable factor in OA development [3]. A recent meta-analysis summarized the 81 unique potential risk factors for developing knee OA following a traumatic knee injury [4], but commonly used drugs for OA were not included. Increasing age is a well-recognized risk factor for OA, affecting an estimated 10% of men and 18% of women over 60 years of age [5]. Additionally, lifestyle factors such as diet and physical activity significantly impact OA progression and symptomatology [2]. Therapeutic strategies often focus on symptom management, where nonsteroidal anti-inflammatory drugs (NSAIDs) like IBU play a crucial role due to their effectiveness in reducing pain and inflammation [6].

IBU is a widely used NSAID that inhibits cyclooxygenase (COX) enzymes, reducing the synthesis of pro-inflammatory prostaglandins [7]. While effective in alleviating pain and inflammation, IBU’s toxic effects on the gastrointestinal tract [8,9], cardiovascular system [10], and kidneys [7] cannot be ignored. Although IBU provides pain relief for OA, there is no evidence that it can halt disease progression. Most OA patients use IBU or other NSAIDs at various stages to manage pain, yet the causal relationship between IBU use and OA progression remains unclear due to potential confounding factors in observational studies.

The etiology of OA is multifactorial, involving genetic, environmental, and metabolic components [11]. To better understand the causal relationships among these factors, Mendelian randomization (MR) provides a robust methodological framework to investigate causal relationships by using genetic variants as instrumental variables, thereby mitigating confounding and reverse causation biases inherent in observational studies [12]. Two-sample MR extends this approach by utilizing summary-level data from separate genome-wide association studies (GWASs) for the exposure (IBU) and the outcome (OA), enhancing the statistical power and accuracy of causal inference [13,14].

This study aimed to explore the causal relationship between IBU use and the risk of developing OA using a two-sample MR approach. By identifying single nucleotide polymorphisms (SNPs) associated with IBU metabolism and action from GWAS as instrumental variables and examining their association with OA in a separate GWAS dataset, we sought to provide robust evidence on the potential causal effect of IBU on OA. This approach aimed to clarify the role of IBU in OA management and inform clinical practices regarding its long-term use.

## 2. Materials and Methods

### 2.1. Data Sources and Selection of Genetic Variants

The datasets utilized in this study were sourced from the IEU OpenGWAS project (https://gwas.mrcieu.ac.uk/, accessed on 1 May 2024). IBU was selected as the exposure, with the most recent GWAS ID ukb-b-8888 (8888) extracted for analysis. OA was chosen as the outcome, with four distinct GWAS IDs extracted: ebi-a-GCST90038686 (90038686), ebi-a-GCST90013881 (90013881), ebi-a-GCST007091 (007091), and ebi-a-GCST005814 (005814), to enhance the reliability of the results. Detailed information regarding the exposure and outcome datasets is provided in Table 1.

### 2.2. Statistical Analysis for MR

MR analysis requires that genetic variants be associated with the exposure but not with potential confounders [15]. To identify SNPs associated with IBU, we applied a threshold of *p* < 5 × 10^−6^, r^2^ < 0.001, clumping distance = 10,000 kb to reduce the impact of linkage disequilibrium (LD).

An F statistic (F = beta^2^_exposure/SE^2^_exposure) greater than 20 was used to minimize bias from weak instrumental variables (IVs) [16]. A threshold of F < 10 has been used to define a ”weak IV” [15]. Therefore, weak instrument bias was negligible. The association between each selected SNP and the risk of OA was then examined (Table S2). Finally, two-sample MR was performed to estimate the causal effect of IBU on OA, utilizing summary statistics from different GWASs. An Odds Ratio (OR) and 95% Confidence Intervals (CIs) obtained from methods of inverse–variance weighted and weighted median estimator were subjected to meta-analysis. All analyses were conducted using R software (version 4.4.1) with the “TwoSampleMR” and “meta” packages.

### 2.3. Estimation of the Causal Relationship between IBU and OA

Five key statistical methods were employed to investigate the causal relationship between IBU and OA: inverse–variance weighted (IVW) [15], MR–Egger regression [17], weighted median estimator [18], Simple_mode and Weighted_mode [19], each with its unique strengths and assumptions. The IVW method combines effect estimates from multiple genetic variants within a meta-analysis framework, weighting them by the inverse of their variance. This method is efficient but relies on the assumption that all genetic variants are valid instrumental variables, which, if violated, could introduce bias. MR–Egger regression accounts for potential directional pleiotropy by incorporating an intercept term in the model; a non-zero intercept indicates pleiotropy, while the slope provides an adjusted causal estimate. Though MR–Egger adjusts for pleiotropy, it may require larger sample sizes and may be less precise. The weighted median estimator offers robustness, providing a valid causal estimate even if up to 50% of the genetic variants are invalid instruments. Simple_mode and Weighted_mode are non-parametric approaches that relax assumptions about instrument validity further. These methods identify the most frequent (modal) value among causal estimates, with Weighted_mode assigning more weight to estimates with lower variance. While robust to multiple invalid instruments, these mode-based estimators may be less efficient than IVW under ideal conditions.

### 2.4. Heterogeneity and Sensitivity Tests

Heterogeneity between SNPs was assessed using Cochran’s Q-statistics and I^2^ (I^2^ = (Q − df)/Q) statistic [20,21,22]. Additionally, a “leave-one-out” analysis was performed to investigate the potential influence of individual SNPs on the causal association.

## 3. Results

### 3.1. Instrumental Variables for MR

**Figure 1:**
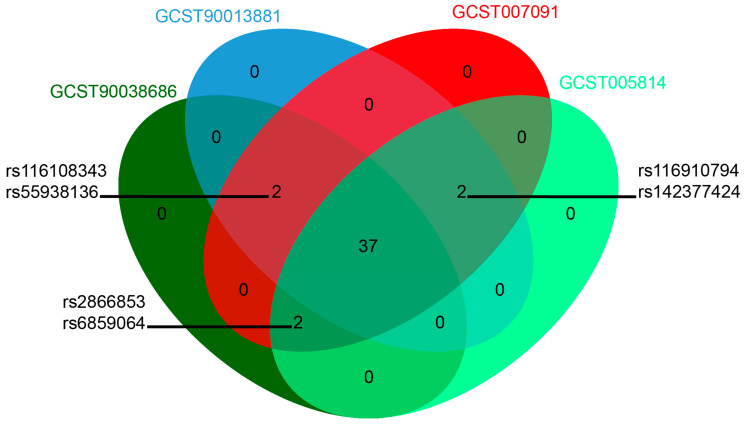
Venn diagram of SNPs from four different GWAS IDs.

We identified 41, 41, 43, and 41 SNPs as instrumental variables for 90038686, 90013881, 007091, and 005814, respectively. In each outcome, 19/41, 17/41, 22/43 and 17/41 SNPs were positively associated with OA, although almost all of them were not statistically significant (Table 2; Table S1). The genetic variants serving as IVs explained 0.06% of variance in the exposure (value of R2 statistic) (Table S2). Interestingly, 37 SNPs overlapped among the four outcomes (Figure 1). The datasets 90038686, 90013881, and 007091 shared the SNPs rs116108343 and rs55938136. Similarly, 90013881, 007091, and 005814 shared the SNPs rs116910794 and rs142377424. Additionally, 90038686, 007091, and 005814 shared the SNPs rs2866853 and rs6859064 (Figure 1). Detailed information about all the SNPs involved in the analyses for each outcome is provided in Table S1.

### 3.2. MR and Meta-Analysis Results

**Figure 2:**
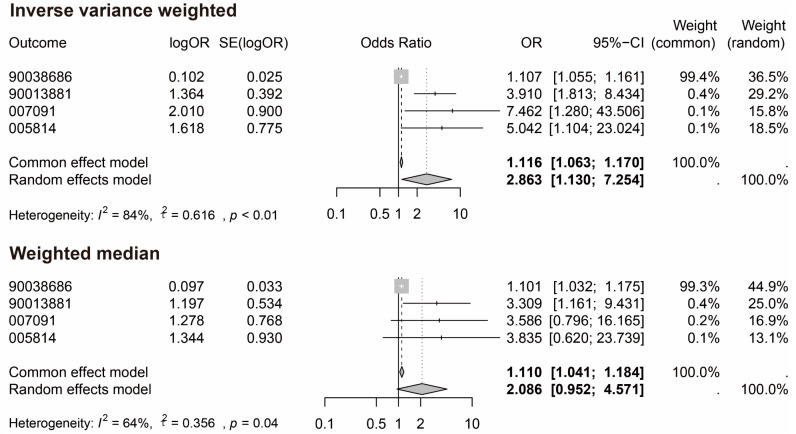
Meta-analysis forest plots for inverse variance weighting (IVW) and weighted median methods.

The study found multiple evidence to support a causal association between IBU and OA in MR analysis combined with meta-analysis and multiple corrections, especially when using the IVW and weighted median methods (Figure 2). For the outcome 90038686, the IVW result produced an OR = 1.107 (95% CI = 1.055–1.161, *p* = 3.5 × 10^−5^), indicating that IBU significantly increases the risk of OA. The weighted median method also supported this association with an OR = 1.101 (95% CI = 1.032–1.175, *p* = 0.004) (Figure 2). For the 90013881, the IVW yielded an OR = 3.910 (95% CI = 1.813–8.434, *p* = 0.001), suggesting that IBU may significantly increase the risk of certain types of OA, particularly in this dataset, where the impact of IBU appears more pronounced (Figure 2). For the 007091, the IVW result showed an OR = 7.462 (95% CI = 1.280–43.506, *p* = 0.001), demonstrating a strong causal relationship between IBU and hip OA. Although the *p*-value for the weighted median method was slightly above 0.05, it still indicated a moderate association (OR = 3.586) (Figure 2). For the 005814, the IVW method’s OR = 5.042 (95% CI = 1.104–23.024, *p* = 0.037), confirming the causal relationship between IBU and OA. The consistency across different methods was generally good, particularly between the IVW and weighted median methods. The direction of association was consistent across all datasets, highlighting the reliability of these methods in causal inference (Table 2). The meta-analysis of IVW results across all datasets demonstrated a common effect model OR = 1.116 (95% CI = 1.063–1.170) and a random effect model OR = 2.863 (95% CI = 1.130–7.254) (Figure 2). Similarly, the meta-analysis of weighted median results showed a common effect model OR = 1.110 (95% CI = 1.041–1.184) and a random effect model OR = 2.086 (95% CI = 0.952–4.571) (Figure 2). The combined OR exceeding 1 further confirmed that IBU is a risk factor for OA.

**Figure 3:**
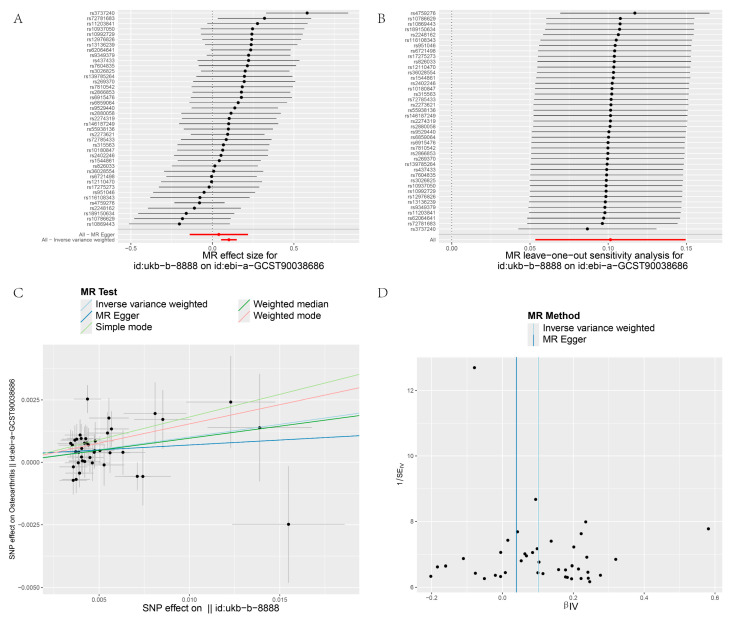
MR analysis IBU on OA (8888 on 90038686): (**A**) forest plot of SNPs associated with IBU and the risk of OA. Black points represent the log Odds Ratio (OR) for OA per standard deviation (SD) increase in IBU, with each SNP treated as a separate instrument. Red points indicate the combined causal estimate using all SNPs together via the MR–Egger test and IVW method. Horizontal lines denote 95% CI. (**B**) Leave-one-out analysis of SNPs associated with IBU and their risk of OA. Each black point represents the IVW MR estimate for the causal effect of IBU, with red points depicting the estimate using all SNPs. No SNP strongly influences the overall effect in this sensitivity analysis. (**C**) Scatter plots of genetic associations with IBU against genetic associations with OA. The slopes of the lines indicate the causal association for each method: the IVW estimate (blue line), MR–Egger estimate (dark blue), simple mode (green), weighted median estimate (dark green), and weighted mode (red). (**D**) Funnel plot assessing heterogeneity. The blue line and dark blue line represent the IVW estimate and MR–Egger estimate, respectively.

**Figure 4:**
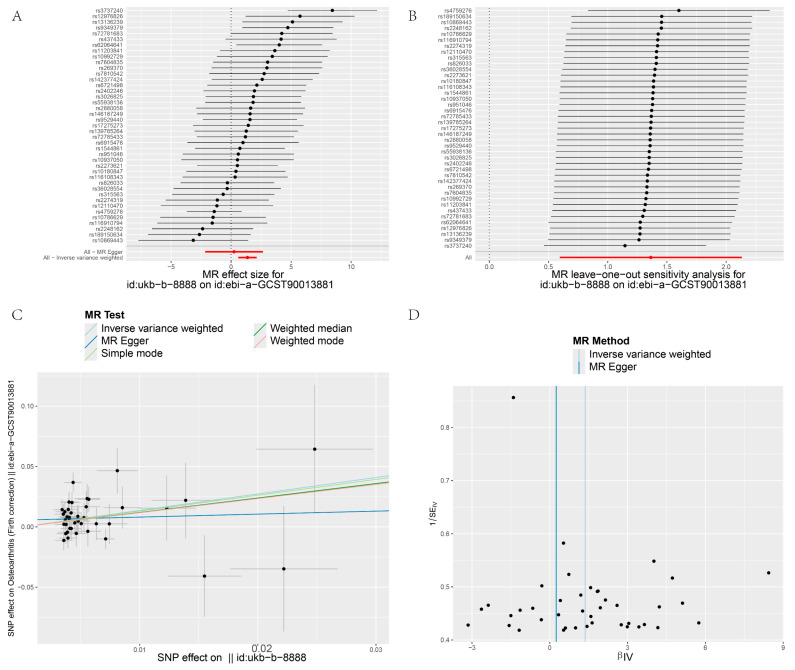
MR analysis IBU on OA (8888 on 0013881). (**A**) Forest plots; (**B**) leave-one-out sensitivity analyses; (**C**) Scatter plots of genetic associations; (**D**) Funnel plots assessing heterogeneity.

**Figure 5:**
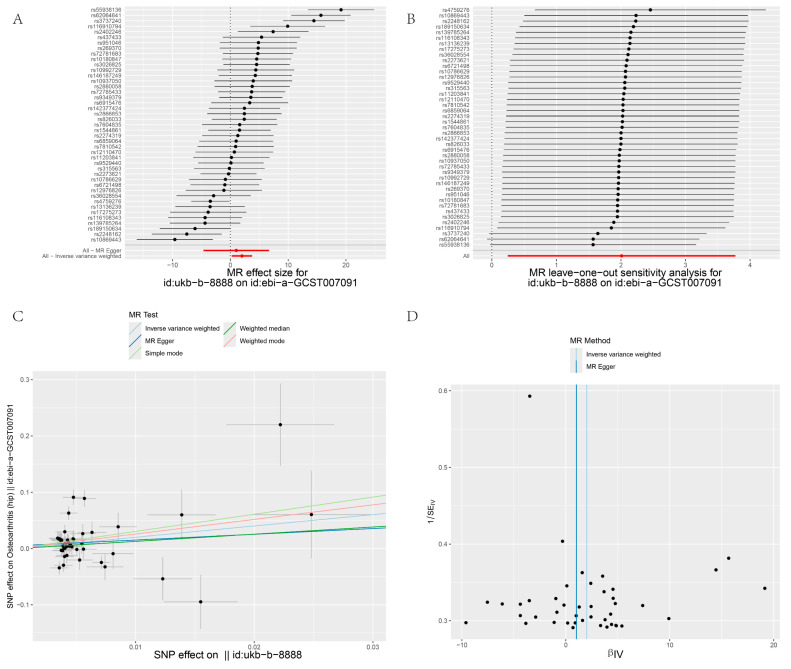
MR analysis IBU on OA (8888 on 007091). (**A**) Forest plots; (**B**) leave-one-out sensitivity analyses; (**C**) Scatter plots of genetic associations; (**D**) Funnel plots assessing heterogeneity.

**Figure 6:**
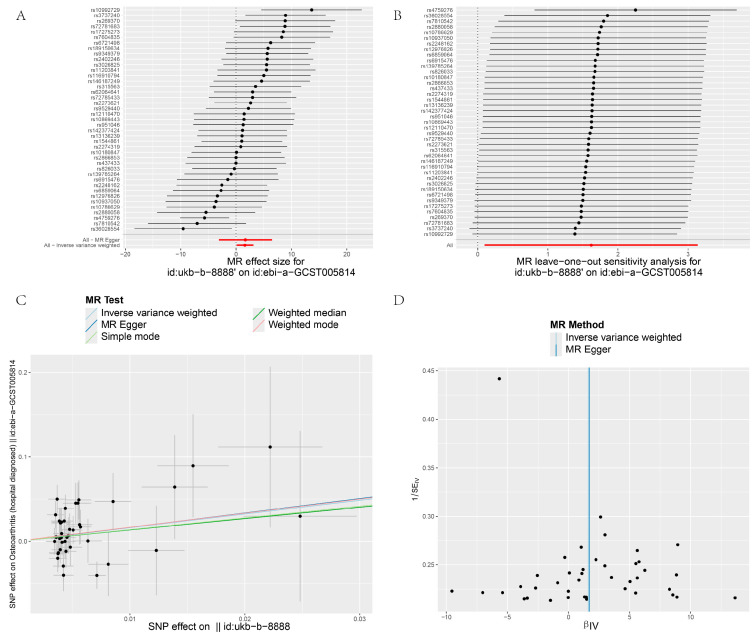
MR analysis IBU on OA (8888 on GCST005814). (**A**) Forest plots; (**B**) leave-one-out sensitivity analyses; (**C**) Scatter plots of genetic associations; (**D**) Funnel plots assessing heterogeneity.

Figure 3, Figure 4, Figure 5 and Figure 6 display the results of MR analyses across four different datasets, the annotations are basically the same. In each figure, panel A shows forest plots, panel B presents leave-one-out sensitivity analyses, panel C provides scatter plots of genetic associations, and panel D shows funnel plots assessing heterogeneity. Specific details can be found in annotation of Figure 3.

### 3.3. Pleiotropy

Testing for pleiotropy is essential to ensure the validity and reliability of MR analysis, as it can introduce bias into causal inferences [15,22]. An intercept that differs from zero (MR–Egger test) is indicative of directional pleiotropy [17]. The results of MR–Egger regression from all outcomes were as follows: for dataset 90038686, intercept = 3 × 10^−4^; *p* = 0.485; for dataset 90013881, intercept = 5.9 × 10^−3^; *p* = 0.343; for dataset 007091, intercept = 4.9 × 10^−3^; *p* = 0.721; and for dataset 005814, intercept = −4.1 × 10^−4^; *p* = 0.972. These intercepts and *p*-values suggest that directional pleiotropy was unlikely to be biasing the result (Table 2). However, the low-heterogeneity *p*-values in the 0013881 and 007091 datasets indicate potential bias from specific SNPs.

### 3.4. Heterogeneity and Sensitivity Test

Heterogeneity refers to the variability in causal estimates across SNPs, with low heterogeneity indicating more reliable MR estimates [22]. Dataset 90038686 exhibited low heterogeneity, enhancing the reliability of its MR estimates (Table 2). In contrast, datasets 007091 and 005814 showed high Cochran’s Q statistics and I² values, indicating substantial heterogeneity and suggesting varied effects of different SNPs (Table 2). To assess the impact of heterogeneity and potential bias, we employed a "leave-one-out" analysis and symmetry funnel plots to detect directional horizontal pleiotropy. For dataset 0038686, despite low overall heterogeneity and pleiotropy, rs4759276 and rs3737240 emerged as potential sources of bias (Figure 3B), with the funnel plot showing slight directional pleiotropy (Figure 3D). In dataset 90013881, the same SNPs were identified as sources of heterogeneity, with slight directional pleiotropy confirmed (Figure 4B,D), Dataset 007091 revealed rs4759276, rs3737240, rs62064641, and rs55938136 as heterogeneity sources, with funnel plots also indicating slight pleiotropy (Figure 5B,D). Lastly, for dataset 005814, rs4759276 was identified as the sole source of heterogeneity, with no evidence of directional pleiotropy (Figure 6B,D).

## 4. Discussion

The causal relationship between NSAIDs like IBU and OA has been a topic of ongoing debate [23,24] despite NSAID’s widespread use for relieving OA symptoms [2]. Our findings provide substantial evidence supporting a potential causal association between IBU use and an increased risk of OA, suggesting that while IBU may offer temporary symptom relief, it could potentially accelerate OA progression, which is consistent with previous research [24,25]. In our study, we used an MR approach to explore the causal relationship between IBU use and OA risk. This approach allows us to minimize confounding factors and reverse causation, providing a more robust inference of causality than observational studies [26]. In contrast, the study by Perry et al. [25] and Driban et al. [24] focused on the association between the current use of medications, including NSAIDs, and the progression of radiographic knee OA using data from the OAs initiative. While their study identified an association between NSAIDs and increased loss of joint space width, it was limited by its observational design, which is prone to confounding and reverse causality.

IBU has been widely used to alleviate the symptoms of OA [27,28]. However, its impact on OA progression remains controversial [23,29]. Several mechanisms may explain the potential detrimental effects of IBU on joint health. First, IBU has been reported to potentially exert negative effects on articular cartilage by inhibiting the metabolic activity of chondrocytes, particularly the synthesis of proteoglycans in cartilage cells [23,30]. Second, long-term NSAID use has also been associated with increased radiographic progression of hip and knee OA [30], likely due to the inhibition of cartilage matrix synthesis. In our study, similar MR analysis results were observed for another NSAID, diclofenac, further supporting the plausibility of these mechanisms. Third, by inhibiting the COX pathway [31], IBU reduces prostaglandin E2 production, which in turn may inhibit cartilage matrix protein synthesis by promoting the release of inflammatory cytokines like interleukin-1 [23]. Additionally, IBU may alter the composition and viscosity of synovial fluid, affecting joint lubrication and load distribution, thus increasing the risk of OA progression.

Clinical studies have yielded mixed results regarding the effects of IBU on OA. For instance, a study within the OA initiative indicated that current users of NSAIDs, particularly IBU, exhibited increased joint space narrowing compared to non-users, suggesting a potential for accelerated joint damage [25]. Conversely, some NSAIDs (IBU) have shown no significant acceleration in radiographic damage over shorter periods of use, indicating that the effects might vary depending on the specific NSAID and duration of use [32]. Given these findings, while IBU is effective for short-term symptom relief in OA, its potential role in promoting long-term OA progression requires careful consideration. Clinicians should prescribe IBU at the lowest effective dose for limited durations, closely monitoring patients for signs of worsening joint health.

Our MR analysis yielded OR values greater than 1 across all outcome datasets using the IVW method, with *p*-values less than 0.05 indicating a significant association between IBU use and an increased risk of OA. Notably, the datasets 90013881 and 007091 produced OR of 3.91 and 7.462, respectively, suggesting that IBU may have a more pronounced impact on specific types of OA, such as hip OA. The consistency of the weighted median estimator results with IVW findings further corroborates the causal relationship between IBU and OA risk.

In assessing heterogeneity and pleiotropy, some datasets (such as 007091 and 005814) exhibited high Cochran’s Q statistics and I² values, indicating substantial heterogeneity. However, the overall heterogeneity was low, enhancing the reliability of the MR estimates. MR–Egger regression results showed intercepts close to zero with large *p*-values, suggesting minimal pleiotropic bias. Our findings were further validated through leave-one-out analysis and funnel plot analysis, which indicated that potential sources of heterogeneity and pleiotropy had limited impact on the overall results.

Despite the robust evidence provided by this study, certain limitations must be acknowledged. The genetic instruments used in our analysis explained only a small proportion of the variance in IBU use, which may limit the precision of our estimates. Additionally, the datasets utilized were derived from populations of European ancestry, which may restrict the generalizability of our findings to other ethnic groups. Future research should focus on replicating these findings in more diverse populations and exploring the underlying biological mechanisms that link IBU use to OA development.

## 5. Conclusions

The results of our MR analysis support a potential causal association between IBU use and an increased risk of OA. Our findings suggest that while IBU may provide symptom relief, it could also contribute to the progression of OA. These results may offer new insights into the mechanisms by which IBU influences the development of OA, highlighting the need for cautious use of this medication in clinical practice.

## Figures and Tables

**Table 1:** Details of GWAS IDs involved in this study.

GWAS ID	Year	First Author	Population	Sample Size	SNP Size	Trait
ukb-b-8888	2018	Ben Elsworth	European	457,547	9,851,867	Medication for pain relief, constipation, heartburn: IBU
ebi-a-GCST90038686	2021	Dönertaş HM	European	484,598	9,587,836	OA
ebi-a-GCST90013881	2021	Mbatchou J	European	407,746	11,039,204	OA (Firth correction)
ebi-a-GCST007091	2019	Tachmazidou I	European	393,873	29,771,219	OA (hip)
ebi-a-GCST005814	2018	Zengini E	European	50,508	15,845,511	OA (hospital diagnosed)

**Table 2:** MR estimates from IVW and MR–Egger of assessing the causal effect of IBU on the risk of OA.

Outcomes	MR Method	SNPs	Beta	OR	Association	Cochran’s Q Statistic	I^2^	Heterogeneity	Pleiotropy
					*p*-Value			*p*-Value	*p*-Value
GCST90038686	IVW	41	0.101	1.107	0	48.75	0.179	0.162	
	MR–Egger	41	0.04	1.04	0.667	48.137	0.19	0.15	0.485
GCST90013881	IVW	41	1.364	3.91	0.001	57.139	0.3	0.039	
	MR–Egger	41	0.246	1.279	0.842	55.822	0.301	0.039	0.343
GCST007091	IVW	43	2.01	7.462	0.025	158.762	0.735	0	
	MR–Egger	43	1.022	2.778	0.726	158.265	0.741	0	0.721
GCST005814	IVW	41	1.618	5.042	0.037	58.671	0.318	0.029	
	MR–Egger	41	1.699	5.47	0.492	58.669	0.335	0.022	0.972

## Data Availability

Publicly available datasets were analyzed in this study. GWAS summary data were downloaded from the IEU Open GWAS project (https://gwas.mrcieu.ac.uk/, accessed on 1 May 2024). IBU was selected as the exposure, with the most recent GWAS ID ukb-b-8888 extracted for analysis. OA was chosen as the outcome, with four distinct GWAS IDs extracted: ebi-a-GCST90038686, ebi-a-GCST90013881, ebi-a-GCST007091, and ebi-a-GCST005814 to enhance the reliability of the results.

## Supplementary Materials

The following supporting information can be downloaded at https://www.mdpi.com/article/10.3390/biology13090748/s1. Table S1: Detailed information on the four selected SNPs; Table S2: The association between each SNP and the risk of osteoarthritis.

## Abbreviations

OA: Osteoarthritis; NSAID: Nonsteroidal Anti-Inflammatory Drug; IBU: Ibuprofen; MR: Mendelian Randomization; GWAS: Genome-Wide Association Studies; SNPs: Single Nucleotide Polymorphisms; OR: Odds Ratio; CI: Confidence Interval.
